# An AI-assisted, failure mode-based toolkit for proactive risk management in radiotherapy: A feasibility study

**DOI:** 10.1016/j.tipsro.2026.100404

**Published:** 2026-04-17

**Authors:** Anastasia Sarchosoglou, Ioannis Genitsarios, Natalia Silvis-Cividjian, Periklis Papavasileiou, Athanasios Bakas, Nikos Papanikolaou, Evangelos Pappas

**Affiliations:** a Department of Biomedical Sciences, Radiology & Radiotherapy Sector, University of West Attica. Athens, Greece; b Department of Radiation Oncology, German Oncology Center, Limassol, Cyprus; c Department of Computer Science, Faculty of Science, Vrije Universiteit Amsterdam, The Netherlands; d Department of Radiation Oncology, Division of Medical Physics, Mays Cancer Center, UT Health-MD Anderson, San Antonio, TX, USA; e School of Biomedical Engineering & Imaging Sciences, Faculty of Life Sciences and Medicine, King’s College London, London, United Kingdom

## Abstract

**Background and purpose:**

Proactive risk management is essential for safe radiotherapy (RT), but formal approaches such as Failure Modes and Effects Analysis (FMEA) can be difficult to implement routinely because of time and resource constraints. We developed i-SART (Intelligent Safety Assistant for Radiotherapy), an AI-assisted, failure mode–based toolkit to support proactive risk management and safety activities in RT, and conducted a feasibility evaluation.

**Materials and methods:**

i-SART is a web-based prototype combining a harmonised failure mode (FM) database derived from 10 FMEA studies, an online portal for FM submissions and a conversational assistant (chatbot) configured to provide structured FM analyses, mitigation suggestions and illustrative incident examples. Internal testing assessed response structure, accuracy and hallucinations. An online survey of RT professionals explored usability and perceived value.

**Results:**

The database contained 419 FMs spanning the RT workflow. The assistant produced structured, clinically plausible mitigation suggestions but was only partially accurate, with hallucinations mainly affecting guideline references and incident details. Fifty-one professionals from 11 countries completed a survey, giving median ratings of 4/5 across domains, including ease of use, support for proactive risk management, and perceived error-reduction potential.

**Conclusion:**

This feasibility study shows that a failure mode–based toolkit combining a curated database, submission portal, and conversational assistant is technically achievable and was rated favourably by early users for supporting risk management activities. Further work should expand the database, strengthen safeguards against hallucinations, and evaluate effects on risk assessment processes and patient safety.

## Highlights

• AI-assisted toolkit (i-SART) supports proactive risk management in radiotherapy.
• Harmonised failure mode (FM) database, FM submission portal and LLM assistant in one toolkit.
• Assistant delivers structured FM analyses aligned with safety guidelines; citation inaccuracies occur.
• Submission portal captures additional prospective risks reported by clinical users.
• Multinational survey shows good usability and perceived value for safety work.

## Introduction

Radiotherapy (RT) is a highly regulated medical discipline. However, incidents can still occur that may compromise treatment outcomes. International safety standards [1] and accreditation schemes [2] therefore emphasise the importance of risk management, while regulatory frameworks require measures to minimise unintended exposures, including risk management integration within RT quality assurance systems [3].

Risk management includes organisational structures and processes that aim to improve patient safety by minimising risks and their consequences through proactive risk assessment and reactive analysis of incidents and near misses [4]. Reactive risk management in RT is commonly implemented through Incident Learning Systems (ILS), which enable reporting and analysis of incidents and near misses to support organisational learning [5]. However, these systems address risks only after they occur and are often limited by underreporting [6]. Proactive methodologies complement ILS [4], [7], enabling earlier identification of potential process failures before patient impact. Rapid technological evolution in RT further increases the need for systematic identification of such failures [1], [8], [9]. Despite regulatory and policy expectations, adoption of proactive risk management methods in healthcare remains limited [10] and uneven in routine RT practice [11], [12], [13], [14], [15]. Time, staffing, expertise, and resource constraints make such approaches difficult to implement, particularly in smaller or resource-limited departments [7], [11], [12], [15]. These challenges are amplified when introducing complex RT techniques without prior institutional experience [15], [16]. The World Health Organization has emphasised the importance of proactively identifying healthcare risks and disseminating this knowledge [17], yet tools for sharing proactive risk information remain limited.

Failure Modes and Effects Analysis (FMEA) is a proactive methodology used to identify, analyse and prioritise potential failures in products, processes or systems before they occur. The AAPM TG-100 report [18] provides guidance for applying FMEA to RT workflows by breaking processes into discrete steps, identifying potential failure modes (FMs), analysing their causes and effects, and scoring severity, occurrence, and detectability to prioritise mitigation strategies. The resulting tabulated FM lists provide a basis for developing harmonised databases that could be shared across institutions [19]. However, such repositories also create a need for intuitive ways for multidisciplinary teams to explore and discuss those risks.

Recent advances in generative AI, particularly large language models (LLMs), offer new opportunities to interact with complex information [20], [21]. In RT, AI tools have mainly been applied to image analysis and treatment planning workflows [22], while early studies suggest LLMs can assist with domain-specific questions and education [23], [24]. However, recognised limitations such as hallucinations and data-governance concerns mean these tools require careful evaluation before clinical use [21], [25].

To address barriers to implementing proactive risk management and support timely sharing of risk information, we developed i-SART (Intelligent Safety Assistant for Radiotherapy), a toolkit combining a harmonised FM database, an FM submission portal and an LLM assistant to support team-based safety activities (e.g., safety huddles, process walk-throughs, FMEA discussions) and individual learning. Preliminary elements of this work were presented at scientific meetings [26], [27], [28]. This study describes the finalised i-SART system and evaluates its technical performance and perceived usefulness for supporting proactive risk management among RT professionals from multiple countries.

## Methods

### Platform Overview

i-SART is a web-based prototype toolkit (https://i-sart.eu/) designed to support proactive risk-management activities in RT, aligned with the AAPM TG-100 [18] approach to FMEA. It provides a structured FM database accessed through an online interface, where users can search, filter and submit FMs, and an LLM assistant that offers context-aware support for safety discussions, suggested mitigation strategies and links to incident examples. When the assistant is launched, the interaction occurs in a separate browser session that displays a standard limitations-of-use notice. A disclaimer on the i-SART landing page indicates the prototype is advisory in nature and non-prescriptive. The design supports iterative updates as clinical needs evolve.

### Technical design

**Fig. 1:**
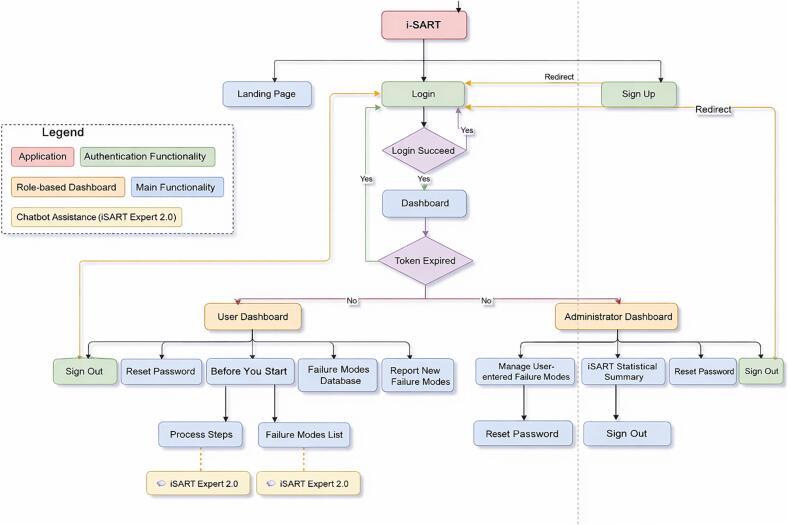
Workflow of the i-SART platform showing user navigation and system functions. Adapted from Ref. [27].

**Fig. 2:**
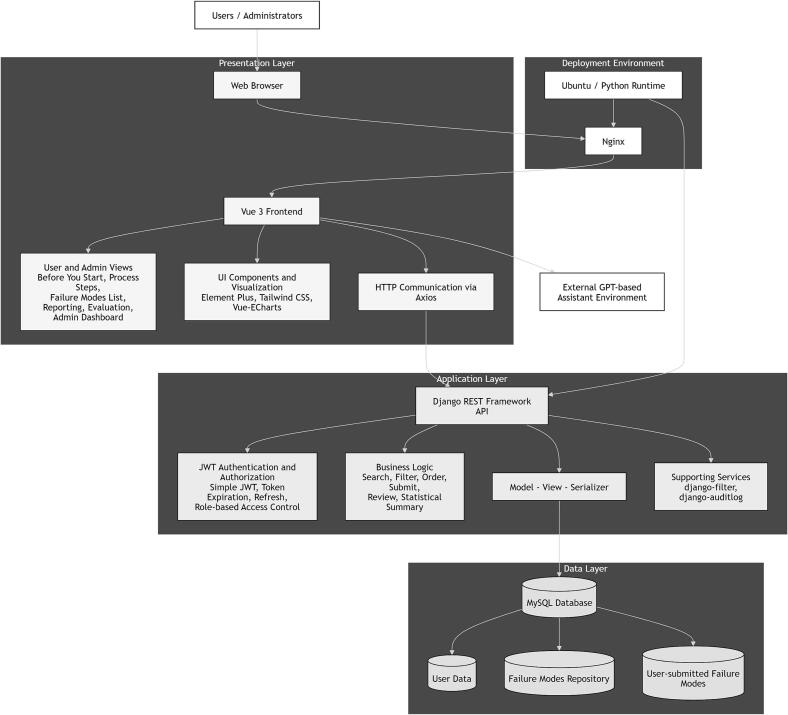
System architecture of i-SART.

The front-end was developed using Vue 3 (Element Plus component library, Vue-ECharts) and Tailwind CSS frameworks. The back-end was developed using the Python/Django REST Framework with a MySQL relational database. Authentication is JSON Web Token (JWT)–based. Role-based access supports Users (search/filter/view FMs; submit FMs) and Administrators (FMs management). The deployment environment is Ubuntu 20.04 with Python 3.10 and Nginx. A workflow diagram of i-SART is shown in Fig. 1, and the system architecture is illustrated in Fig. 2.

### Failure mode Database: Sources and structure

The i-SART database currently aggregates FM entries from eight published and two unpublished FMEA studies and related methodologies such as Failure Mode, Effects and Criticality Analysis (FMECA) and Healthcare Failure Mode and Effect Analysis (HFMEA). These sources cover external-beam modalities, including 3DCRT [29], IMRT [18], VMAT [30], SGRT [31], SRS/SRT [19], SBRT (unpublished), MR-guided adaptive radiotherapy (MRgART) [32], [33], and the general RT pathway [34]. Eligibility criteria included English-language, diversity of RT techniques, representation of multiple countries to capture variation in clinical practice, and sufficient detail for standardised mapping.

**Fig. 3:**
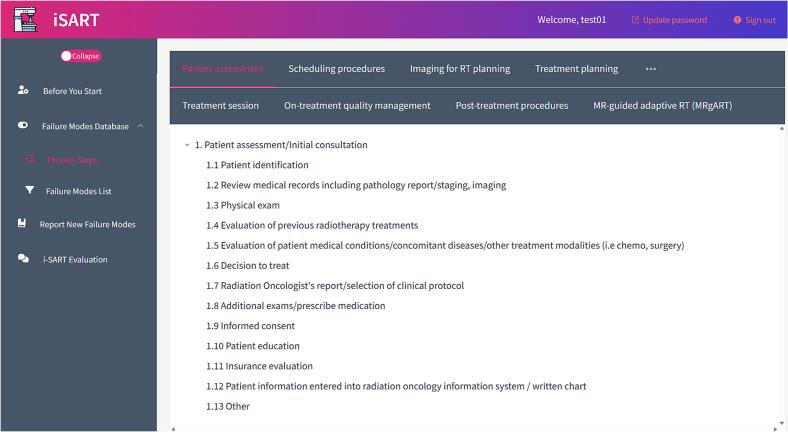
I-sart interface showing process-map navigation across radiotherapy subprocesses.

A process map defines eight standard RT subprocesses with 61 steps (Fig. 3). To reflect its technique-specific workflow, MRgART is treated as a separate subprocess (12 steps) and displayed as a distinct tab in the subprocess ribbon.

**Fig. 4:**
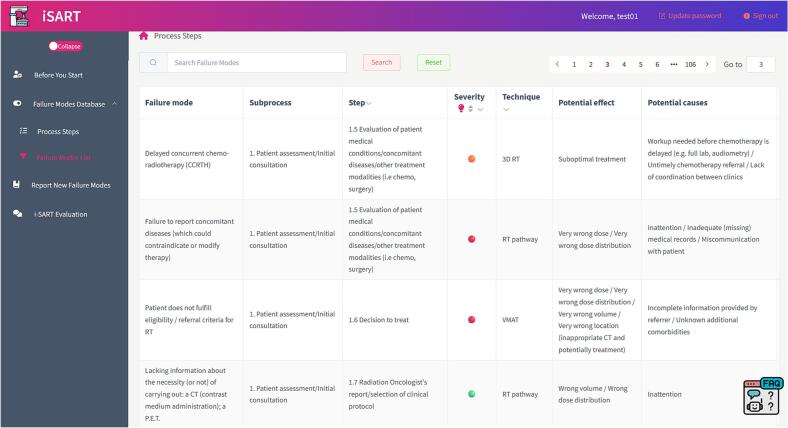
Example entries from the i-SART failure mode database.

Each FM is mapped to a subprocess, process step, technique, severity, effects, and causes, as exemplified in Fig. 4.

**Fig. 5:**
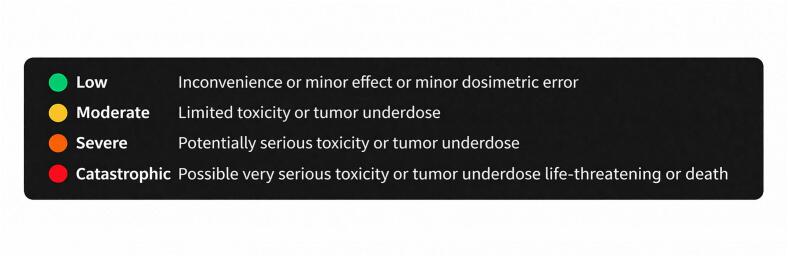
Four-tier severity scale used in i-SART.

Severity was classified on a four-tier, colour-coded scale for rapid prioritisation (Fig. 5). This scale was derived by harmonising the severity scales used in the source FMEA/HFMEA/FMECA studies into a single system (Supplementary S1.1).

While the underlying data model accommodates detectability and occurrence of FMs, these indices are not displayed at this stage because current data are limited and inconsistent, which would risk misleading generalisations and cross-institutional comparisons. Instead, severity was retained as the primary metric visible to users, providing an indication of potential consequences. The system design preserves the ability to incorporate additional indices in future iterations as the corpus expands.

Preprocessing workflow: FM text was cleaned and normalised, followed by Natural Language Processing-supported duplicate detection (explicit, implicit, and hierarchical variants) and expert review for semantic validation before inclusion in the database (Supplementary Material S1.2).

### User interface

The i-SART interface enables navigation of the users through a hierarchical process map and searchable tables with filters for step, technique, and severity or keywords. Users can submit new entries via an online structured form and provide feedback through an integrated survey page. A dedicated button launches the i-SART Expert 2.0 chatbot, offering on-demand, context-aware support for FMEA, safety queries, and mitigation strategies.

### Chatbot design

i-SART Expert 2.0 is a GPT-4o–based LLM chatbot configured for RT FMEA support. It uses prompt engineering rather than model fine-tuning, with a fixed expert persona and document-prioritised logic emphasizing AAPM TG-100. As GPT-4o is a proprietary, pre-trained model, training/validation partitioning and hyper-parameter disclosure are not applicable; reproducibility is instead supported through full reporting of prompt logic and curated database provenance.

User queries are interpreted based on clinical context and process step, drawing on guidance from TG-100, mapped mitigation strategies from international RT guidelines, and incidents from a curated knowledge base comprising reports from major organisations (IAEA/SAFRON, ASTRO-AAPM/Ro-ILS, UKHSA, ASN, ARPANSA, CPQRC). Responses follow a structured template comprising TG-100 analysis, mitigation strategies, and illustrative incident examples. In this prototype, retrieval was facilitated through the GPT knowledge-file mechanism rather than a separately engineered external vector database pipeline. Measures intended to mitigate hallucination risk include the use of a curated source corpus, document-prioritised prompt logic emphasising TG-100, a structured response format, and explicit labeling of synthetic incident examples. The chatbot supports multilingual interaction through a secure browser interface and can be launched directly from i-SART for context-specific risk analysis. A representative prompt-and-reply transcript is provided in Supplementary Material S2.1.

### In-house chatbot testing

The i-SART Expert 2.0 chatbot was initially tested with about 20 prompts to identify typical error patterns and guide prompt refinements. From these, a subset of seven representative test cases (four mitigation-focused prompts and three incident-focused prompts) was selected for structured evaluation and documentation. For each test case, the full response, including both the substantive content and its references, was reviewed as a single unit. Responses were classified as accurate, partially correct or incorrect, and as hallucination present or absent, with particular attention to citation fidelity and the presence of invented or over-specific details. The final seven cases are summarised in the Results section (Chatbot performance) and Supplementary Material S2.2.

### i-SART user evaluation

We conducted a multinational online survey inviting RT professionals (radiation therapists, medical physicists, radiation oncologists and safety/quality managers) to assess i-SART, using convenience sampling via professional networks and social media. The primary endpoint was usability. Secondary endpoints covered perceived assistant effectiveness and clinical relevance (supporting proactive risk management, raising safety awareness, strengthening safety culture and knowledge gain), perceived potential for error reduction, overall satisfaction and ease of entering FMs. Items were rated on five-point Likert scales with one open-text question. Respondents also reported role, years of experience, familiarity with proactive risk management and country of practice. The full questionnaire, including item wording and response anchors, is provided in Supplementary S1.3. Participation was voluntary and anonymous, with electronic informed consent obtained at the start of the survey. No patient data or personal identifiers were collected, and, per institutional policy, formal ethics approval was not required. Because recruitment used professional networks and social media, a response rate could not be determined. Analyses were descriptive, reporting counts, percentages and medians with interquartile ranges.

### Data Security

i-SART is intended for non-patient-specific use. No patient data or protected health information were used in platform development or evaluation. Data transmitted between client and server travels over encrypted channels. Authentication uses JSON Web Tokens with short-lived sessions.

## Results

### Failure modes database

**Fig. 6:**
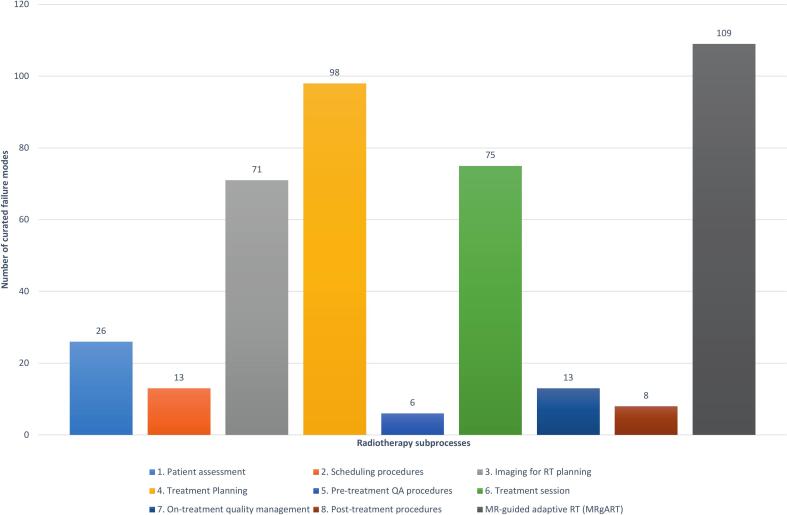
Distribution of curated failure modes (n = 419) across radiotherapy subprocesses, including MR-guided adaptive RT (MRgART).

The initial corpus of 728 FM was curated to 419 unique entries, each mapped to RT subprocesses, steps, and techniques for streamlined access. The curated FMs were not uniformly distributed across subprocesses. The largest proportions were related to treatment planning and treatment session. MRgART contributed a significant and distinct subset of FMs (Fig. 6). In i-SART, MRgART was treated as a separate subprocess, rather than being distributed across the standard RT subprocesses, due to its distinct MR-guided workflow and quality-assurance processes.

During the evaluation period, users submitted 14 additional FM, several of which were not previously present in i-SART.

Examples included:

• Incorrect SGRT surface selected (e.g., phase 1 instead of phase 2/boost).
• Scheduling external beam radiotherapy and brachytherapy on the same day.

### Chatbot performance (In-house Testing)

Seven representative prompts (four mitigation-focused and three incident-focused) were used to assess the behaviour of the i-SART Expert 2.0 assistant in safety-related conversations. All test cases were reported as partially correct with the presence of hallucinations. The assistant produced structured responses in which the mitigation suggestions were judged clinically plausible and broadly consistent with TG-100 principles and RT safety guidelines. However, none of the responses were fully accurate when evaluated as a whole. Across the seven prompts, hallucinations were observed, most commonly as fabricated or over-specified references to guideline sections and embellished details in illustrative incident narratives. An illustrative example is shown in Box 1. Full prompt/response logs are provided in Supplementary Material S2.2.

Box 1 **Example of the chatbot response (Test Case 1: Omission of Pacemaker Reporting).**

**Table:** 

**Prompt:**	*“FM: Omission of reporting the presence of pacemaker, what are the mitigation strategies?”*

**i-SART Expert 2.0 Chatbot Response** (summary):	Standardized intake forms, checklist verification, electronic medical record alert, team communication, and staff training (with TG-100 section citations).
**Appraisal:**	Mitigation strategies were clinically plausible and aligned with TG-100 and safety principles (accurate core content), but the cited TG-100 sections were incorrect or over-specified, and some details were presented as if directly sourced from TG-100 when they were not (hallucinated citations). Therefore, the response was rated as partially correct with hallucinations present.

### User evaluation

**Fig. 7:**
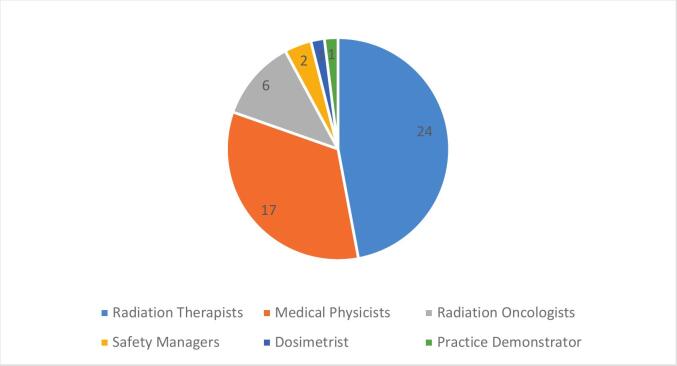
Distribution of professional roles among survey respondents (n = 51).

A total of 51 professionals from 11 European countries completed the survey; the distribution by role is shown in Fig. 7. Most respondents (62 %, 32 out of 51) reported more than ten years of experience, and 47 % (24/51) were very or extremely familiar with proactive risk assessment, with the remainder reporting at least some familiarity.

Ratings were generally favourable (median 4/5 across domains; IQR typically 3–4 or 4–4**;** Table 1). Among respondents who reported using the assistant in a language other than English (n = 29), 28/29 (96.6 %) rated the assistant as effective or very effective. Open-text feedback (n = 49) praised the intuitive use, training value, and safety focus, with requests for database expansion, mobile optimisation, and tighter linkage to incident-learning systems and citations.

**Table 1:** Survey domains with median (1–5) and interquartile range (IQR, Q1–Q3); higher scores indicate more favourable ratings. Total respondents N = 51.

Survey domain	N	Median (1–5)	IQR(Q1-Q3)
Ease of use	51	4	4–4
Chatbot effectiveness	51	4	4–4
Raising safety awareness	51	4	4–4
Risk management utility	51	4	4–4
Error reduction potential	51	4	4–4
Strengthening safety culture	51	4	4–4
Knowledge gain	51	4	3–4
Overall satisfaction	51	4	4–4

## Discussion

In this feasibility study, we developed i-SART, a prototype web-based toolkit that combines a curated FM database, an FM submission portal, and an LLM assistant to support proactive risk management and patient safety practices in RT, and conducted a preliminary evaluation. The design of i-SART was driven by the aim of providing RT teams and individual practitioners with a practical toolkit for proactive safety work, usable both in safety meetings and for self-directed learning. The database offers rapid access to structured descriptions of process risks, the assistant supports exploration of those risks through natural-language interaction in multiple languages, and the submission portal enables users to contribute additional potential FMs for knowledge sharing.

The curated database demonstrates one way to reuse existing FMEAs beyond their original local context. Building the harmonised FM database required integrating material from FMEA, HFMEA and FMECA studies that used differing terminology and severity scales; FM descriptions were cleaned, de-duplicated and mapped to a common process map, and severity scores were harmonised into a single system. Although this required non-trivial curation, the process shows that heterogeneous FMEA outputs can be consolidated into a usable FM database spanning the RT workflow. The resulting distribution of FMs was not uniform: most entries related to treatment planning and treatment delivery. This pattern likely reflects the focus and granularity of the source studies but is broadly consistent with previous RT FMEA reports [19], [35], most likely because these stages are technically complex and involve multiple parameters. Earlier workflow stages may be less frequently represented in published analyses, although they may still involve important latent risks. The separation of MRgART in the database illustrates that emerging technologies introduce additional workflow steps and associated risks and may therefore require periodic review to identify additional FMs. Quantifying the true risk profile of different subprocesses was not the aim of this project, but the current coverage appears sufficient, at this early stage, to support exploratory FMEA-style activities and discussions. The FM submission portal complements the curated content by providing a low-threshold way for users to document potential FMs that may not be captured in published FMEAs or in ILS. During the evaluation period, a subset of participants used the portal to submit 14 additional FMs, some of which were not present in the database. Although this number is small, it indicates that some users were willing to engage with the feature and to share locally observed risks, and illustrates how a toolkit like i-SART could, in the future, complement ILS by capturing prospective process risks.

The internal assistant testing provides an initial view of how a domain-configured LLM behaves in safety-related conversations. In this in-house evaluation, the assistant’s responses were well structured and the mitigation suggestions were judged clinically plausible and aligned with established safety principles, but the overall accuracy was limited by citation drift and over-specific details, with hallucinations mainly affecting guideline references and incident cases. These patterns are consistent with broader evaluations of hallucinations in natural language generation systems [21], [36]. In its current configuration, outputs are informational and non-prescriptive and are intended to support discussion, brainstorming and training rather than clinical decision-making, consistent with published guidance on the ethical use of AI in healthcare [37], [38]. Nonetheless, sound clinical judgement remains essential. Because FMs represent only one element of the broader FMEA process, which also requires multidisciplinary evaluation, risk prioritisation and implementation of mitigation strategies within departmental quality-management programs, i-SART is intended to support proactive safety discussions rather than replace established risk-management processes.

Survey responses suggest that RT professionals can use this type of toolkit and perceive clear added value. Across respondents from multiple roles and countries, ratings were consistently favourable, with median scores of 4/5 across domains including ease of use, support for proactive risk management, safety awareness, assistant effectiveness and perceived potential for error reduction and knowledge gain. Free-text comments indicated that users appreciated the structured, workflow-based view of FMs and saw i-SART as a practical aid for risk-management activities and safety education, while also calling for expanded content, better mobile usability and improved support for cross-checking chatbot outputs against source guidelines.

Previous work by Kornek et al. has described a digital risk-management system for RT with department-level FMEA templates, quantitative prioritisation of FMs and integration into clinical workflows [39], [40]. i-SART shares this overall goal of facilitating proactive risk management but differs in emphasis and scope, focusing on qualitative exploration of process risks. i-SART is primarily intended as a departmental support tool for safety discussions, exploratory FMEA-style activities and training. At a broader level, its harmonised FM database, community contribution features and the assistant could support knowledge sharing across institutions and complement national and international incident-learning initiatives. It may also contribute to greater consistency in the identification and discussion of safety risks, particularly for emerging RT techniques where collective experience is still evolving.

Data governance and deployment context are crucial considerations for any AI-assisted tool. i-SART is intentionally limited to non-patient-specific use; it is not connected to clinical information systems, and users are instructed not to enter protected health information or identifiable institutional details. Even so, the use of a third-party LLM API means that prompts and responses may be processed outside institutional infrastructure. For the present feasibility work, restricting use to research and educational contexts is appropriate, but any move towards institutional deployment would need to follow established guidance on data governance and acceptable use for AI in healthcare [37], [38].

As this was a preliminary feasibility work, several limitations should be noted. The FM database is limited to a sample of FMEA studies and is influenced by the selection of those sources; it does not yet incorporate all published FMEAs or data from incident-learning databases. The assistant’s outputs are not yet systematically organised using formal human factors/ergonomics or systems-safety methods. The internal assistant evaluation was qualitative and restricted to seven test cases, so it provides only an initial view of assistant behaviour. The user survey was convenience-sampled, with a modest sample size, so the findings reflect preliminary perceptions rather than generalisable attitudes. This study also did not assess objective outcomes such as error-detection rates or time required for risk-analysis tasks, and cannot make claims about clinical impact.

These limitations point to priorities for future work. On the content side, expanding and regularly updating the FM database, particularly for emerging techniques, will be essential. Future work should examine how knowledge base expansion influences assistant performance, including whether additional curated domain data (e.g., FMs, incident reports, and guidelines) improves completeness, consistency, grounding, and generalisability, or whether increased corpus heterogeneity introduces ambiguity, conflicting evidence, or reduced precision. Performance could be compared across progressively expanded corpora.

On the technical side, future work on the assistant will explore domain-adapted approaches using curated domain data to improve response faithfulness and controllability. Given that hallucinations in this study mainly affected guideline references and incident cases, future guardrails should particularly target citation verification, provenance display, and stricter separation of source-supported content from synthetic content. We also plan to structure outputs using a work-system human factors framework (e.g., Systems Engineering Initiative for Patient Safety [SEIPS] [41]) and to explore systems-theoretic hazard analysis methods such as System-Theoretic Process Analysis (STPA) [42], [43]. In this first feasibility prototype, we deliberately adopted a simpler, prompt-based configuration of a general-purpose LLM, domain-configured for RT safety, to test usability and perceived usefulness before investing in more complex architectures. Future studies should also include task and outcome-based evaluations to assess clinical impact.

## Conclusion

In this feasibility study, we developed and evaluated i-SART, a prototype toolkit that integrates a harmonised failure-mode database, a failure-mode submission portal and a large-language-model assistant to support proactive risk management in radiotherapy. We demonstrate the technical feasibility of aggregating failure modes from multiple FMEA studies into a workflow-indexed resource, capturing additional user-submitted risks via a submission portal, and providing AI-assisted conversational support for risk analysis discussion and training. Multinational survey responses from RT professionals indicated good usability and perceived value, while internal assistant testing highlighted both the potential and the limitations of LLM-based assistance in this setting. The toolkit is primarily intended for departmental use but may also support knowledge sharing at national and international levels. Future work should expand the database, strengthen safeguards against hallucinations, assess how knowledge base expansion affects assistant performance and evaluate the toolkit’s impact on risk analysis processes.

## Funding

This study received no specific funding for the research. The article processing charge (APC) is supported by the Special Account for Research Grants (ELKE) of the University of West Attica.

## Declaration of competing interest

The authors declare that they have no known competing financial interests or personal relationships that could have appeared to influence the work reported in this paper.
