# Supplementary material for: An AI-assisted, failure mode-based toolkit for proactive risk management in radiotherapy: A feasibility study
Source: Tech Innov Patient Support Radiat Oncol. 2026 Apr 17;38:100404. doi: 10.1016/j.tipsro.2026.100404 (PMC13146522; doi:10.1016/j.tipsro.2026.100404)
Supplement: Supplementary Data 1 [file mmc1.docx]

# Supplementary S1 — Methods Supplement

Contents: S1.1 Severity scale harmonization; S1.2 Failure mode (FM) data cleaning and normalization; S1.3 Survey instrument and codebook.

## S1.1 Severity scale harmonization

Several severity scoring systems have been applied in radiation therapy (RT) risk assessments (Table S1.1), including:

- Healthcare Failure Mode and Effects Analysis (HFMEA, 1–4 scale)
- Simplified FMEA/FMECA adaptations (1–5 scale), and
- AAPM TG-100 framework (1–10 scale)

To enable uniform visualization of severity across the i-SART FM database, heterogeneous source scales were harmonized to a four-tier scale (Low, Moderate, Severe, Catastrophic - Table S1.2) and displayed with a traffic-light color scheme for visual cues. This unified mapping supports **consistent interpretation** across published methodologies (e.g., FMEA, HFMEA, simplified FMEA/FMECA, AAPM TG-100).

**Table S1.1**. Severity Scales in RT Risk Assessment

| Score | HFMEA (1–4) | Simplified FMEA/FMECA (1–5) | TG-100 (1–10) |
| --- | --- | --- | --- |
| 1 | Minor: No injury or minimal inconvenience | Negligible: No harm, no outcome impact | No effect |
| 2 | Moderate: Increased length of stay or additional treatment, no permanent harm | Minor: Temporary inconvenience, minimal impact | Inconvenience |
| 3 | Major: Permanent lessening of function, disfigurement, surgical intervention required | Moderate: Reversible injury, treatment delay, moderate impact | Inconvenience |
| 4 | Catastrophic: Death or major permanent loss of function | Major: Serious permanent injury or significant impact | Minor dosimetric error, suboptimal plan or treatment |
| 5 | – | Catastrophic: Death or complete failure | Limited toxicity or tumor underdose |
| 6 | – | – | Limited toxicity or tumor underdose (greater severity) |
| 7 | – | – | Potentially serious toxicity or tumor underdose |
| 8 | – | – | Possible very serious toxicity or tumor underdose |
| 9 | – | – | Possible very serious toxicity or tumor underdose (greater severity) |
| 10 | – | – | Catastrophic |

**Table S1.2**. Harmonized Mapping

| i-SART Color | i-SART Classification | HFMEA (1–4) | Simplified FMEA (1–5) | TG-100 (1–10) |
| --- | --- | --- | --- | --- |
| Green | Low: Inconvenience, minor effect, or minor dosimetric error | 1 | 1–2 | 1–4 |
| Yellow | Moderate: Limited toxicity or tumor underdose | 2 | 3 | 5–6 |
| Orange | Severe: Potentially serious toxicity or tumor underdose | 3 | 4 | 7–8 |
| Red | Catastrophic: Very serious toxicity, life-threatening event, or death | 4 | 5 | 9–10 |

**Methods Note on Harmonization**

AAPM TG-100 assigns score 4 to minor dosimetric errors. For harmonization, we classified this level as Low (green), reasoning that toxicity or tumor underdose is only described from TG-100 score 5 onwards.

## S1.2 Failure‑mode data cleaning and normalization

This document outlines the curation process used to standardize and deduplicate 728 FMs prior to integration into the i-SART platform. A combined approach involving semi-automated processing and manual expert review was employed to enhance consistency and remove redundancies. The process involved the following steps:

**1. Text Normalization**

All FM entries were processed using standard text-cleaning techniques, including:
Lowercasing; Removal of trailing punctuation; Expansion of abbreviations; Presence of non-English characters. Entries deemed malformed were excluded from the dataset during this phase.

**2. Workflow-Based Grouping**

To improve contextual accuracy, FMs were first grouped by their associated RT subprocess (e.g., imaging, simulation, planning, delivery). Deduplication was applied separately within each RT subprocess to ensure that context-specific FM were not mistakenly merged, even if they appeared similar in wording.

**3. Duplicate Detection**

Duplicate FMs were identified and resolved in three conceptual categories:

A. Explicit Duplicates

Entries that were identical in the same step.

B. Implicit Duplicates

*Duplicates Based on Keywords*

Entries expressing the same meaning with different wording (e.g., paraphrases). These were identified using Natural Language Processing (NLP)-based keyword matching, with morphological variants generated by Python libraries such as 'inflect' and 'word-forms'. Due to error-prone automation, final manual verification was performed.

Example of keyword matching:

| Sub-process | Step | Keywords |
| --- | --- | --- |
| 6. Treatment planning | 6.4 Delineation of target(s) and organs at risk | wrong, incorrect, poor, imperfect |
|  |  | contouring, delineation, outline, exams, PTV, CTV, GTV, target, organs at risk |

Example of FM duplicates:

- “Oncologist outlines target incorrectly”
- “Wrong target contouring”

C. Hierarchical Duplicates

Records that shared a structural prefix (e.g., 'Specify ROI for optimization process: …'). These were flagged through structural parsing and subsequently validated manually.

**Example:**

| Failure Mode | Subprocess | Step | Severity (1-10) | Source /  Technique | Effect | Causes |
| --- | --- | --- | --- | --- | --- | --- |
| 1. Specify ROI for optimization process: Incorrect ROI volumes | 6. Treatment planning | 6.6 Treatment plan production | 6.78 | TG100 / IMRT | Wrong dose | Inadequate contour capping algorithm |
| 2. Specify ROI for optimization process: Incorrect ROI volumes | 6. Treatment planning | 6.6 Treatment plan production | 6.89 | TG100 / IMRT | Wrong dose | Improper handling of branching structures |
| 3. Specify ROI for optimization process: ROI expansion outside or close to the outer skin contour | 6. Treatment planning | 6.6 Treatment plan production | 5.56 | TG 100 / IMRT | Suboptimal plan | Algorithm limitations |

Entries were merged when all the post-colon descriptor denoted the same FM (i.e., substantively identical wording/intent); severity values were comparable (within the same tier after harmonization); effects and causes were compatible (no contradictions). Entries were not merged when the descriptor introduced a distinct FM.

Accordingly, FMs 1 and 2 were merged, while FM 3 was retained as a separate entry. The screenshot S1.1 illustrates how FMs 1 and 2 now appear as a single FM in i-SART.


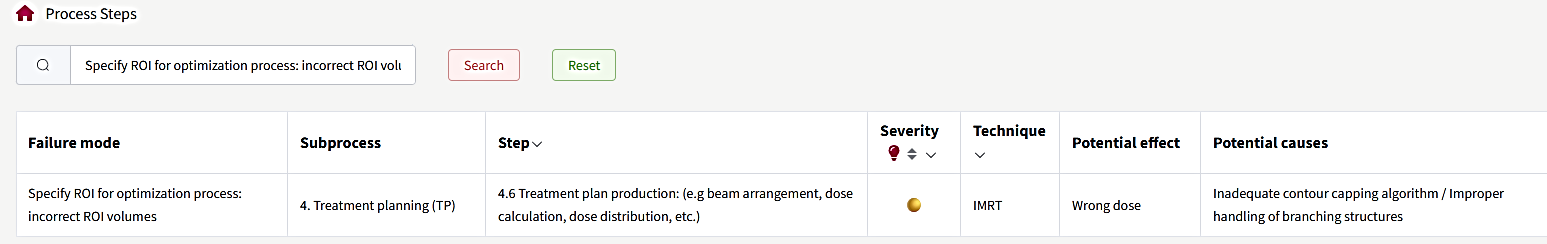


**Figure S1.1** Screenshot of the failure mode “Specify ROI for optimization process: Incorrect ROI volumes”.

**4. Manual Semantic Validation**

All FM were manually reviewed to confirm semantic equivalence before removal or consolidation. This ensured clinical accuracy and preserved meaningful distinctions between similar but non-identical FMs.

**5. Pre-processing Outcome**

After normalization, exclusion, and deduplication, 419 unique FMs were retained from the original 728. The final dataset captures diverse risks across the RT workflow while eliminating redundancy and improving semantic consistency.

This curated dataset was then inserted into the database and supports the platform’s ability to provide relevant, searchable potential FM.

**S1.2.1 Source corpus (published FMEA/FMECA studies)**

The harmonized FM dataset was assembled from eight published and two unpublished FMEA studies spanning multiple radiation therapy techniques (e.g., 3DCRT, IMRT, VMAT, SGRT, SRS/SRT, SBRT, MRgART, and the general RT pathway). Full citations are listed in S1.4 (Supplementary References, Methods) as S1‑R1–S1‑R8.

**Technique cross‑walk:** IMRT — S1‑R1; 3DCRT — S1‑R2; VMAT — S1‑R3; SGRT (DIBH breast) — S1‑R4; SRS/SRT — S1‑R5; MRgART — S1‑R6–S1‑R7; General RT pathway — S1‑R8.

## S1.3 Survey instrument and codebook

This supplement reproduces the survey items, response scales, and collection context used for the i‑SART user evaluation.

**Fielding window:** 17 Dec 2024 – 21 May 2025

**Instrument language**: English

**Response scales (5‑point Likert)**

Scale 1: Very Difficult | Difficult | Neither Difficult nor Easy | Easy | Very Easy

Scale 2: Very Ineffective | Ineffective | Neither Effective nor Ineffective | Effective | Very Effective

Scale 3: Not Useful at All | Slightly Useful | Neither Useful Nor Not Useful | Very Useful | Extremely Useful

Scale 4: Strongly Disagree | Disagree | Neither Agree nor Disagree | Agree | Strongly Agree

Scale 5: Strongly Disagree | Disagree | Neither Disagree nor Agree | Agree | Strongly Agree

Scale 6: Very Unlikely | Unlikely | Neither Unlikely nor Likely | Likely | Very Likely

Scale 7: Very Unsatisfied | Unsatisfied | Neither Unsatisfied nor Satisfied | Satisfied | Very Satisfied

**Likert‑type items**

| Item label | Question text (column header) | Response scale |
| --- | --- | --- |
| Ease of use | Ease of Use: How easy is it to use the i-SART tool? | Very Difficult \| Difficult \| Neither Difficult nor Easy \| Easy \| Very Easy |
| Chatbot effectiveness | Chatbot Effectiveness: How effective is the i-SART chatbot at assisting with your queries? | Very Ineffective \| Ineffective \| Neither Effective nor Ineffective \| Effective \| Very Effective |
| Non‑English effectiveness | How effective was the i-SART chatbot when you used it in a language other than English? | Very Ineffective \| Ineffective \| Neither Effective nor Ineffective \| Effective \| Very Effective |
| Entering a failure mode (ease) | How easy was it to enter a failure mode into i-SART? | Very Difficult \| Difficult \| Neither Difficult nor Easy \| Easy \| Very Easy |
| Raising safety awareness | Raising Safety Awareness: How useful do you find i-SART in raising awareness about safety within radiotherapy practices? | Not Useful at All \| Slightly Useful \| Neither Useful Nor Not Useful \| Very Useful \| Extremely Useful |
| Error reduction potential | Impact on Error Reduction: Do you believe that i-SART has the potential to reduce errors in the workplace? | Strongly Disagree \| Disagree \| Neither Agree nor Disagree \| Agree \| Strongly Agree |
| Risk management utility | Assisting with Proactive Risk Management: How useful do you find i-SART in assisting with proactive risk management? | Not Useful at All \| Slightly Useful \| Neither Useful Nor Not Useful \| Very Useful \| Extremely Useful |
| Strengthening safety culture | Strengthening Safety Culture: Do you believe that i-SART can strengthen safety culture by improving the understanding of risks in workflows and tasks among your colleagues? | Strongly Disagree \| Disagree \| Neither Disagree nor Agree \| Agree \| Strongly Agree |
| Knowledge gain | Impact of i-SART on Professional Knowledge: Do you believe that i-SART has helped you gain new knowledge applicable to your work regarding patient safety? | Strongly Disagree \| Disagree \| Neither Disagree nor Agree \| Agree \| Strongly Agree |
| Likelihood to recommend | Likelihood of recommendation: How likely would you be to recommend i-SART to others? | Very Unlikely \| Unlikely \| Neither Unlikely nor Likely \| Likely \| Very Likely |
| Overall satisfaction | Overall Satisfaction: Considering that i-SART is currently in its pilot phase and ongoing improvements are expected, how satisfied are you with the tool so far? | Very Unsatisfied \| Unsatisfied \| Neither Unsatisfied nor Satisfied \| Satisfied \| Very Satisfied |

**Demographic items**

| Item | Response options (observed) |
| --- | --- |
| Role | Radiation Oncologist, Medical Physicist, Radiation therapist / Radiotherapy Radiographer / Therapeutic Radiographer / Radiotherapy technologist), Practice demonstrator, Dosimetrist, Safety / Quality Manager |
| Years of experience | 6-10 years, 1-5 years, More than 10 years |
| Familiarity with proactive risk management | Somewhat Familiar , Very Familiar, Extremely Familiar, Slightly Familiar, Not At All Familiar |
| Country | Responses from: Greece, Italy, Portugal, Albania, Malta, Serbia, Slovenia, Bosnia and Herzegovina, United Kingdom, Belgium, Ireland |

## Notes

• One open‑text item invited comments on usability and clinical relevance.

• Conditional item: non‑English effectiveness was shown to respondents who reported using the assistant in a non‑English language.
