# Supplementary material for: An AI-assisted, failure mode-based toolkit for proactive risk management in radiotherapy: A feasibility study
Source: Tech Innov Patient Support Radiat Oncol. 2026 Apr 17;38:100404. doi: 10.1016/j.tipsro.2026.100404 (PMC13146522; doi:10.1016/j.tipsro.2026.100404)
Supplement: Supplementary Data 2 [file mmc2.pdf]

Supplementary S2 — Results Supplement

Contents: S2.1 Representative chatbot prompt, reply transcript; S2.2 Chatbot testing logs (setup, rubric, seven-case summary, notes, aggregate observations).

S2.1 Representative chatbot prompt - reply transcript

Structured FMEA-based safety discussion on the i-SART failure mode (FM): "Inaccurate CBCT-CT registration".

Prompt 1: FMEA Analysis of "Inaccurate CBCT-CT Registration"

User Prompt:
Can you walk me through an analysis for the failure mode 'Inaccurate CBCT-CT registration'?

i-SART Expert 2.0 Response:

Failure Mode: Inaccurate CBCT-CT registration
This means the CBCT acquired for patient setup is misaligned with the planning CT — but the error is either not detected or is accepted mistakenly.

Process Step

- **Image-guided radiotherapy (IGRT)** or **patient setup verification** prior to treatment.
- This occurs after the CBCT image is acquired and before the treatment beam is delivered.

Potential Causes (Causal Factors)

- Poor image quality (e.g., motion artifact, low contrast)
- Misalignment of image datasets (manual or automatic)
- Anatomical changes between simulation and treatment (weight loss, tumor regression)
- User error in selecting landmarks or matching volumes
- Use of incorrect registration parameters or software defaults
- Hardware/software malfunctions (e.g., system lags or bugs)
- Fatigue or workflow pressure (e.g., time constraints)
- Inexperience or inadequate training in manual fusion

Potential Effects

- Geographic miss of the target (underdosing tumor)
- Overdose to adjacent organs-at-risk (OARs) (e.g., spinal cord, rectum, bladder)
- Systematic error if the same misregistration is used over multiple fractions
- Compromised treatment efficacy and increased toxicity risk
- Re-treatment or clinical harm if error is discovered late

TG-100 Reference: Section 3.3 – *Failure Modes and Effects Analysis (FMEA)*; Section 4.4 – *Identification and scoring of failure modes*

Prompt 2: Real-World Incident Description

User Prompt:
Can you describe any real-world radiotherapy incidents related to inaccurate CBCT-CT registration from your incident knowledge base?

i-SART Expert 2.0 Response:

Real-World Incident: Inaccurate CBCT-CT Registration

📌 **Incident Summary**

- **Event Type**: Image registration error — CBCT to planning CT
- **Treatment Site**: Prostate
- **Modality**: IMRT with daily CBCT-based alignment
- **Discovery**: Day 7 offline review by a senior therapist

📖 **What Happened**

During the first seven fractions of a prostate IMRT treatment, the CBCT was consistently misregistered with the planning CT. The auto-match algorithm placed the isocenter too posteriorly, and the treating therapist accepted the alignment without manual correction or second review.

A review of Day 1–7 shifts during a weekly QA meeting showed that:

- The daily shifts were unusually consistent, but not within the expected anatomical range for that patient.
- The prostate was being partially missed in the superior-posterior direction.

The error was caught by a senior therapist during a retrospective offline review of the CBCT series.

⚠ **Consequence**

- Geographic miss of the prostate CTV for 7 consecutive fractions
- Underdosing of the posterior prostate
- Unintended exposure to adjacent tissues
- Reported as a near-miss with clinical consequence, not requiring re-treatment but flagged as a learning event

□ **Contributing Factors**

- Overreliance on auto-match
- Lack of peer review for Day 1 CBCT
- Time pressure in a high-throughput clinic

- No shift threshold warning system in place
- Inadequate image registration training for newer staff

Reference: Real incident from *Incidents.pdf* knowledge base

Prompt 3: Mitigation Strategies for CBCT-CT Registration

User Prompt:
What mitigation strategies can reduce the risk of inaccurate CBCT-CT registration?

i-SART Expert 2.0 Response:

Mitigation Strategies for Inaccurate CBCT-CT Registration:

1. Peer Review of CBCT Registration on Day 1
Independent review of image registration at the start of treatment and at key intervals can help detect systematic errors early. This is especially important for sites with high motion sensitivity or ambiguous anatomy.

2. Automated Shift Threshold Alerts with Stop-and-Review Protocols
Large or unexpected couch shifts may indicate a registration error. Setting pre-defined shift thresholds (e.g., ≥5 mm) can prompt automatic alerts, requiring additional verification before proceeding with treatment.

3. Mandatory Manual Review of Auto-Match Registration Results
Automatic fusion tools can misalign structures, particularly in mobile or low-contrast regions such as the pelvis. Manual inspection of all matches ensures that the alignment is clinically appropriate before couch shifts are applied.

4. Site-Specific IGRT Protocols with Defined Match Criteria and Tolerances
Different anatomical sites require tailored registration strategies. Clearly defined protocols for each site—including what to match on, acceptable variances, and review steps—improve consistency and reduce uncertainty in image alignment.

5. Routine CBCT Image Registration Training and Competency Assessments
Consistent and accurate registration relies on trained personnel. Regular training sessions and documented competency assessments help standardize interpretation and reduce variability among staff.

6. Use of Fusion Overlays and/or Quantitative Match Quality Indicators
Enhanced visualization tools such as color blending, dual-contrast overlays, or match scoring systems help users identify subtle mismatches that may not be apparent on grayscale images alone.

7. Scheduled Retrospective Review of CBCT Registrations and Couch Shifts
Periodic review of registration images and recorded shifts during the treatment course allows

teams to identify deviations, confirm protocol adherence, and catch errors that may have escaped initial detection.

Reference: Supported by *Mitigations.pdf* knowledge base
TG-100 Reference: Section 5 – *Risk Mitigation Strategies*

S2.2 Chatbot testing logs

Purpose: To document an in-house test of the i-SART assistant's responses for accuracy and hallucination patterns, using representative prompts aligned with proactive risk management tasks in radiation therapy. This supplement provides setup, rubric, a summary table of seven test cases (four prompts for mitigation strategies and three for documented (real) incident examples), and brief per-case notes.

1. Test Setup

Model & configuration: GPT-4o via i-SART Expert 2.0; prompt-engineering configuration (no fine-tuning); fixed expert persona; informational, non-prescriptive intent.

Knowledge sources available to the assistant: AAPM TG-100, a curated mitigation document (RT guidelines), and an incidents compendium (e.g., IAEA/SAFRON, ASTRO-AAPM/Ro-ILS, national reports).

Scope & goal: Evaluate whether the assistant provides structured, TG-100-aligned guidance without fabricating citations/incidents, and whether mitigation suggestions are reasonable for the queried FM.

2. Scoring Rubric

Accuracy rating definitions: Correct = factually sound and appropriately sourced; Partial = generally reasonable suggestions but with gaps or mis-attributions; Incorrect = materially wrong or misleading.

Hallucination assessment: Present = fabricated, over-specific, or mismatched citations/incidents; Absent = no fabricated specifics and sources match.

Key failure patterns flagged during review included: (i) citation drift (TG-100 section/table mismatches), (ii) over-specific invented incident narratives, and (iii) over-reach beyond source scope (e.g., naming technologies or guidelines not explicitly present in the cited document).

3. Summary of Test Cases

Case	Prompt theme	Accuracy	Hallucination	Main issue observed
**1**	Pacemaker presence omitted - mitigation request	Partial	Present	Reasonable mitigations; TG-100 section mis-cited; attribution over-reach
**2**	Pacemaker omission - real incident request	Partial	Present	Incident narratives initially framed as real; later acknowledged as fictional
**3**	Wrong patient in room (Day 1) - mitigation request	Partial	Present	Adds technologies not cited in TG-100 (e.g., RFID); TG-100 mis-cites
**4**	Patient ID errors - real incident request	Partial	Present	Blends general themes from incident compendium with invented narrative _detail
**5**	Incorrect interpretation of tumor/normal tissue - mitigation request	Partial	Present	Mitigations largely reasonable; cites consensus/gadgets not in TG-100; mis-cites section
**6**	Prescription after planning - wrong patient/dose/site/plan - mitigation request	Partial	Present	Reasonable checks; claims TG-100 sections/tables inconsistently; adds hard-stops not explicitly cited
**7**	Wrong site treated - real incident request	Partial	Present	One theme supported; other narratives not traceable to source

4. Brief Notes by Case

Case 1. Failure mode - Pacemaker presence omitted – mitigation request: Mitigation suggestions matched standard practice (intake prompts, checklists, EMR flags); however, specific TG-100 sections/tables were mis-cited or over-attributed.

Case 2. Pacemaker omission - real incident request: Assistant provided incident narratives labeled as real; upon challenge, acknowledged they were illustrative. The source document supports the general issue but not the exact narratives.

Case 3. Failure mode - Wrong-patient in the room (day 1) - mitigations request: core mitigations aligned (two identifiers, checklists, time-out); added technologies (RFID/barcoding) and section references not traceable to TG-100.

Case 4. Patient-ID - incident request: incidents presented as real-case summaries; details exceeded what is present in the incident compendium. Themes (e.g., similar names, language barriers) are supported in general.

Case 5. Failure mode - Contouring/interpretation - mitigation request: peer review, checklists, education are supported; explicit citations to RTOG/ESTRO and automation tools were not found in TG-100; section mis-cite.

Case 6. Failure mode - Prescription-related - mitigation request: independent verification, plan–Rx consistency checks align conceptually; claims of specific TG-100 tables/sections and R&V hard-stops were over-specific.

Case 7. Wrong-site - real incident request: laterality themes exist in sources; detailed narratives were not traceable. Over-specificity flagged as hallucination.

5. Aggregate results

Quantitative outcomes:

- Accuracy: 0/7 fully correct; 7/7 partial; 0/7 incorrect.
- Hallucination presence: 7/7 (100%).
- Reviewer concordance: 100% agreement between two independent raters.

Breakdown of hallucination types observed:

- Citation drift or mis-citing TG-100 sections/tables: 4 of 4 mitigation cases.
- Invented or over-specific incident narratives: 3 of 3 incident cases.
- Over-reach beyond source scope (technologies/guidelines not cited in TG-100): 4 of 4 mitigation cases.

Interpretation:

Across the seven prompts, mitigation suggestions were generally aligned with safety principles and TG-100 concepts. However, none of the responses were fully accurate when evaluated as a whole, and all seven were therefore classified as exhibiting some form of hallucination. These hallucinations most commonly involved citation drift or over-specific details (e.g., in illustrative

incident narratives). Despite this, mitigation strategies remained clinically plausible and consistent with safe practice.
